# Hypothetical Control of Heart Rate Variability

**DOI:** 10.3389/fphys.2019.01078

**Published:** 2019-08-23

**Authors:** Bruce J. West, Malgorzata Turalska

**Affiliations:** ^1^Information Sciences Directorate, US Army Research Office, Durham, NC, United States; ^2^Computational and Information Sciences Directorate, CCDC Army Research Laboratory, Adelphi, MD, United States

**Keywords:** heart beat variability, inverse power law, scale invariance, Lévy process, fractional Fokker-Planck equation

## Abstract

In the last three decades, the analysis of heart rate variability by nonlinear methods demonstrated the complexity of cardiovascular regulation. Additionally to the observations of periodic heart rate regulation by the autonomic nervous system, the long-term statistics of the heart rate has been determined to reminisce a tempered Lévy process. A number of heuristic arguments have previously been made to support a tempering conjecture, using exponentially truncated waiting times for the time intervals between heart beats. Herein we use the fractional probability calculus to frame our arguments and to parameterize the control process that tempers the Lévy process through a collective-induced potential. We also determine that the hypothesis of a self-induced nonlinear potential control resulting in such a tempered Lévy process is consistent with the hypothesis of disease being the loss of physiologic complexity made over 25 years ago.

## 1. Introduction

The cardiovascular system forms a complex structure, where nonlinear control mechanisms shape the cardiac activity at the scales of seconds, minutes and hours (Iyengar et al., 1996; Goldberger, 2006). Over the past thirty years, fluctuations in heart rate, referred to as heart rate variability (HRV), have become a central topic in physiological signal analysis, serving as a vital non-invasive indicator of the performance and physiological state of the cardiovascular system. Both decreased statistical and spectral indices of HRV have been linked to poor outcomes in patients with e.g., myocardial infraction (Kleiger et al., 1987), coronary artery disease (Huikuri, 1995) and heart-failure (Guzzetti et al., 2000), while distinct peaks in the power spectrum of HRV have been attributed to autonomic, sympathetic and vagal modulation.

Furthermore the analysis of HRV demonstrated the complexity of cardiovascular regulation, revealing non-linear and non-Gaussian nature of heart rate series (Beckers et al., 2006). Identified nonlinear fluctuations in HRV entailed important physiologic advantages associated with the ability of the cardiovascular system to respond to unpredictable stresses and stimuli, since nonlinear responsiveness offers greater flexibility than a linear reaction (Goldberger and West, 1987; Shlesinger and West, 1991). The work of Peng et al. (1993) was the first of many studies focusing on the scaling properties of HRV time series, showing the spectrum of the inter-beat time increments to scale as an inverse power law (IPL) in frequency:

(1) $$\begin{array}{r} S\left(f\right)\propto \frac{1}{f^{2H-1}}. \end{array}$$

Observed lack of characteristic scale in heart rate fluctuations was attributed to various control processes which occur at different time scales in the cardiovascular system. As a result, the HRV time series fluctuate in a complex, erratic manner in healthy individuals, even those at rest. However, this IPL scaling has a surprising consequence for the behavior of the variance of heart rate time series, suggesting that the second moment of this signal increases with the progression of time. This corresponds to increasing time intervals between consecutive heart beats, which in the long time limit results in an unphysiological condition. We will revisit this puzzling conclusion in details further along in this paper.

The correlations, captured by the spectral analysis, characterizing temporal ordering of beat-to-beat differences, *I*(*n*) = *B*(*n* + 1) − *B*(*n*), where *B*(*n*) denotes the length of heart beats for the beat number *n*, are interpreted to be anti-correlated in normal healthy individuals. Correspondingly, this correlation was determined to vanish in patients with heart disease (Peng et al., 1993). However, the loss of correlation in diseased individuals was determined to not influence the probability density function (PDF) of the heart beat increments. Both healthy and diseased individuals were described by a Lévy stable PDF and it was not possible to statistically distinguish between them. This observation demonstrated that the distribution of inter-beat fluctuations does not uniquely characterize HRV time series, since it is their time ordering that accurately captures the fractal nature of heart beat time series.

The analysis of HRV performed by Peng et al. (1993) indicated that the statistics of the changes in heart beat intervals are Lévy stable. This observation challenged the assumption that cardiac activity observed in healthy individuals is normal in statistical sense. Peng et al. (1993) demonstrated that rather than following a bell curve of Gaussian distribution, statistics of heart beat intervals is characterized by heavy tails, signifying that deviations from the average are more likely than expected from a Gaussian process. However such functional dependence shares an undesirable property with one observed through the HRV spectrum, namely a diverging second moment. This implies that the heart beat of a healthy individual would be asymptotically unstable, a conclusion that is physiologically unacceptable. A number of suggestions have been made to overcome this unrealistic divergence of the HRV. Herein we adapt some of the ideas from control theory as a means of resolving the issue.

The empirical Lévy PDF is at the center of our analysis and suggests that the fractional calculus provides a strategy for going beyond the random walk interpretation of the HRV time series proposed by Peng et al. and accepted by a generation of scientists. The probability that the dynamic variable *I*(*t*) lies in the phase space interval (I,I+dI) at time *t* is P(I,t)dI, where the probability distribution P(I,t) is defined as a solution to the fractional Fokker-Planck equation (FFPE) (West, 2013):

(2) $$\begin{array}{r} \frac{\partial P\left(I,t\right)}{\partial t}=K_{\alpha }\partial_{\left|I\right|}^{\alpha }\left[P\left(I,t\right)\right], \end{array}$$

where we have introduced the symmetric Reisz-Feller fractional derivative $\partial_{\left|I\right|}^{\alpha }\left[\cdot \right],$ whose Fourier transform is −|ω|^α^ and *K*_α_ is a constant. Equation (2) is a fractional diffusion equation for the change in the heart beat intervals, whose solution is given by the symmetrc Lévy distribution (West, 2013):

(3) $$\begin{array}{r} P\left(I,t\right)=\underset{0}{\overset{\infty }{\int }}\cos\left[I\omega \right]\exp\left[-tK_{\alpha }\omega^{\alpha }\right]\frac{d\omega }{\pi }. \end{array}$$

The PDF given by Equation (3) agrees with the histograms of the HRV data (Peng et al., 1993) for fixed time, with α = 1.7 and *K*_α_ > 0. The inclusion of the Fokker-Planck equation allows defining an equation governing the evolution of probability distribution for heart rate intervals. However it's intrinsic value lies in forming the connection between the equation defining the evolution of the heart rate intervals as a time series, the Langevin equation mentioned subsequently, and the equation observing the evolution of its probability distribution. In terms of a random walk-like process, the duality is that of observing evolution of a single walker versus having information about the probability of its location at a given time. The probability framework is thus richer, since it allows for estimation of possible values of heart rate intervals; e.g., prediction of a chance of a specifically long interval between heart beats.

Herein we hypothesize the existence of cardiovascular control mechanism which leads to a tempered Lévy distribution characterizing HRV in healthy individuals. This mechanism suppresses the largest changes in inter-beat intervals, those being in the tails of the Lévy PDF, which persist in the various cardiac disorders. The pathophysiology of the HRV PDF being Lévy stable, therefore results from the suppression of this physiological control process and differs from the exponential tempering of the Lévy PDF found in the literature (West, 2014a) and used by others (Kiyono et al., 2006). We model the HRV as a nonlinear Langevin equation, where the nonlinear terms correspond to the control process. The corresponding Fokker-Planck equation takes a fractional form, whose steady-state solution is a tempered Lévy distribution. To our knowledge this is the first attempt at capturing the dynamics of cardiovascular system though the framework of fractional calculus.

## 2. Control Hypothesis

The heart's beat-to-beat time series, *B*(*n*), is a consequence of numerous control mechanisms that shape the cardiac activity. The most fundamental component of the HRV is formed by the firing of the pacemaker cells in the heart's sinoatrial node (Hall, 2016). This periodic action is continuously modulated by two competing branches of the autonomic nervous system (ANS), with sympathetic stimulation, respectively, increasing, and parasympathetic action decreasing, the firinig rate (Goldberger and West, 1987; Goldberger et al., 1990; Peng et al., 1993). Herein we address the significance of the heart's primary pacemaker, the sinoatrial node, in controlling the balance between these two branches of the autonomic nervous system. The importance of the response of the sinoatrial node to autonomic signaling to heart rate and HRV has been emphasized by Yaniv et al. (2013)

We discuss the regulation of extremes in the intermittent fluctuations of HRV time series following the observations of Kiyono et al. (2004, 2006) of measuring the degree of non-Gaussianity of heart rate fluctuations. This approach is based on quantifying the degree of deviation of empirical data from Gaussianity, originally developed in the study of the intermittent properties of velocity fluctuations in turbulent flow by Castaing et al. (1990). The cascading mechanism that determines the intermittency in turbulence was postulated to give rise to analogous intermittent behavior in HRV time series (Lin and Hughson, 2001). It is worth pointing out that the intermittency observed in turbulent fluid flow can also be described using the fractional calculus to obtain a Lévy PDF (West, 2014a). The diffusion of passive tracers track the velocity fluctuation in heterogeneous media and have been shown to be exponentially tempered to capture the natural cutoff of waiting times (Meerschaert et al., 2014).

Kiyono et al. (2004) and Kiyono et al. (2006) reported robust scale-invariant properties in non-Gaussian distributions observed in healthy human HRV spanning a wide range of temporal scales. However they found that a truncated or exponentially tempered Lévy PDF could not be ruled out as a proper descriptor of the HRV statistics, since sufficient coarse graining of the HRV time series resulted in a Gaussian distribution, implying that the underlying process had a finite variance. Furthermore, in a series of papers the interpretation of the origin of the non-Gaussian properties of HRV has been called into question. Initial proposal connecting the HRV intermittency to the critical state-like dynamics (Kiyono et al., 2004) has been challenged in Kiyono et al. (2006), where PDF of the HRV has been equally well fitted by a tempred Lévy PDF and a log-normal cascade-type multiplicative process (Castaing et al., 1990). On the other hand, the cascading mechanism could not be confirmed in the analysis of HRV by Kiyono and Bekki (2011). This is in conflict with an earlier finding of Lin and Hughson (2001), who found strong evidence that the cascade mechanism can generate some of the statistics of HRV variability. In the following sections we outline a novel interpretation of the above results, one proposing nonlinear control of HRV.

**Figure 1:**
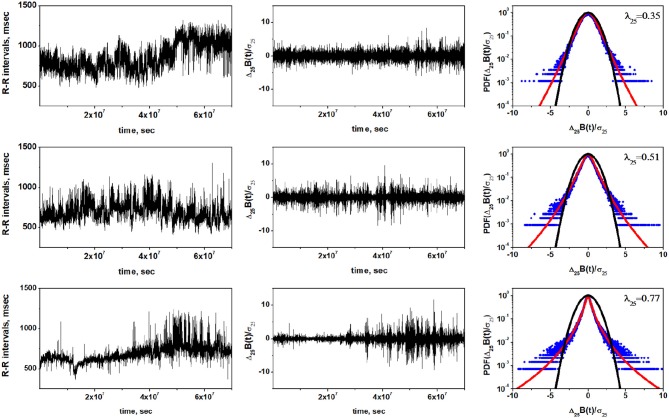
Representative examples of the non-Gaussianity index analysis of the HRV in a healthy individual **(Top row)**, CHF patient of class I, II, III **(Middle row)**, and CHF patient of class III, IV **(Bottom row)**. Left column shows time series of inter-beat intervals, middle column displays corresponding time series of standardized heart rate increments. Right column shows standardized PDFs of heart rate increments. Estimated values of the non-Gaussianity index λ_25_ are show in each PDF panel. In a solid red line we show the PDF approximated by the non-Gaussian model (Equation 22 in Appendix). The black line represents the Gaussian distribution.

We analyze three sets of experimental data of HRV from the MIT-BIH RR Interval Database (Goldberger et al., 2000) in order to illustrate motivations behind the proposed control mechanism of HRV. Information concerning adopted datasets and performed analysis are discussed in details in the Appendix 5.1. The values (mean ± SD) of commonly reported HRV indices are presented in Table 1. Figure 1 shows the heart rate intervals, standardized time series of heart rate increments and standardized PDF of the heart rate increments for a representative examples of individuals belonging to three groups: a healthy individual, a congestive heart failure (CHF) patient of class I, II, III and a congestive heart failure (CHF) patient of class III, IV. In line with results presented in Hayano et al. (2011), the PDFs for the heart beat interval variability have non-Gaussian form, with increased value of the non-Gaussianity index λ observed in progressively worse clinical groups. The difference between healthy individuals, CHF patients of class I, II, III and CHF patients of class III and IV suggests that the last group could be modeled by a Lévy PDF, whereas healthy patients might be modeled by a truncated or tempered Lévy PDF. This interpretation is consistent with the results of Kiyono et al. (2006), but would not have been evident from the limited data available to Peng et al. (1993).

**Table 1:** Measures of heart rate variability in healthy subjects and two groups of patients with congestive heart failure.

		**Healthy**	**CHF**	**CHF**
			**(I, II, III)**	**(III, IV)**
Time domain	Mean RR, *ms*	785 ± 90	807 ± 107	798 ± 127
	SDNN, *ms*	122 ± 29	109 ± 40	86 ± 37
	rMSSD, *ms*	31 ± 15	28 ± 18	22 ± 14
Frequency domain	VLF power, *ms*^2^	1,650 ± 900	1,347 ± 945	836 ± 750
	LF power, *ms*^2^	1,150 ± 534	501 ± 690	389 ± 512
	HF power, *ms*^2^	540 ± 723	268 ± 425	243 ± 275
	LF/HF ratio	2.1 ± 1.2	1.8 ± 1.3	1.6 ± 0.9
Nonlinear measures	DFA α_1_	1.25 ± 0.18	1.09 ± 0.11	0.96 ± 0.13
	DFA α_2_	0.89 ± 0.14	0.85 ± 0.16	0.78 ± 0.12
Non-Gaussianity index	λ_25_	0.33 ± 0.08	0.56 ± 0.11	0.63 ± 0.16

*Values are reported as mean ± SD. SDNN, SD of normal RR intervals; rMSSD, square root of the mean of the squared differences between adjacent normal RR intervals; VLF, very-low-frequency power; LF, low-frequency power; HF, high-frequency power; LF/HF, ratio of low- to high-frequency power; DFA α_1_, short-scale index of detrended fluctuation analysis; DFA α_2_, long-scale index of detrended fluctuation analysis; λ_25_, non-Gaussianity index*.

Motivated by the fundamental role the sinoatrial node plays in maintaining the balance between opposite actions of the autonomous nervous system, we hypothesize that the activity of this node acts as a cardiovascular control mechanism that produces a tempered Lévy PDF in healthy individuals. This activity suppresses the largest changes in heart-beat intervals, ones forming the tails of a Lévy PDF observed in the severe CHF group. The presence of the Lévy distribution in the HRV PDF of pathological cases is thus a result of a disease process which affects and reduces action of the sinoatrial node. Loss of this control mechanism leads to the imbalance between sympathetic and parasympathetic activity of ANS, what has been documented in numerous cardiac diseases (Luo et al., 2018).

In section 3 the mathematical form of the tempered Lévy hypothesis is introduced in the form of a nonlinear potential in a stochastic differential equation to describe HRV dynamics. Note that this model does not correspond to the exponential tempering of the Lévy PDF found in the literature (West, 2014a) and used by others (Kiyono et al., 2006) to account for the observed properties of the HRV time series. In section 4 the control mechanism is interpreted in terms of the disease being the loss of complexity in medicine. Some conclusions are also drawn.

## 3. Hypothetical Control

Control theory is not usually discussed in the physics literature, in part, because it is thought to give an anthropomorphic tinge to the discussion. We believe this is because control is what engineers introduce into a process to obtain a desired outcome, whereas natural selection accomplishes the same task, in terms of enhanced fitness, but with more subtlety, if less intent. Biological macro-evolution selects for the most robust dynamics that emerges out of physical laws. Consequently, we assume a hypothetical dynamic equation for the inter-beat intervals, formally expressed as a nonlinear stochastic differential equation

(4) $$\begin{array}{r} dI\left(t\right)=F\left(I,t\right)dt+d\xi \;(t), \end{array}$$

where *F*(*I, t*) is a deterministic driver and *dξ*(*t*) is a stochastic driver. The forms of the two drivers, the deterministic piece being defined by the dynamics of the sinoatrial node and the random piece being determined by the autonomic signal, are consistent with the arguments of Yaniv et al. (2013) and can be made functionally specific by what is known about HRV time series.

First and foremost, the statistics of the heart beat time series have a non-Gaussian PDF and multi-fractal scaling properties (Kiyono et al., 2004). As mentioned in the Introduction, the HRV time series is the result of competing neuroautonomic inputs. The competition between these two branches of the involuntary nervous system is the mechanism assumed here to provide the erratic variability recorded in healthy subjects (Goldberger and West, 1987; Goldberger et al., 1990) and therefore determines the statistics of the stochastic driver. Thus, we assume the statistics of the autonomic inputs in the inter-beat interval Langevin equation *dξ*(*t*), resulting from the competition between the two neuroautonomic inputs, to be Lévy stable.

Last, but not least, the form of the deterministic driver emerges from the intrinsic dynamic properties of the sinoatrial node pacemaker cells. These properties are modeled herein using the recent observation that biological systems are poised at criticality (Mora and Bialek, 2011), which can in principle produce the cooperative oscillations, as well as erratic variability, observed in rhythmic heart beat time series. However, the fluctuations arising from such chaotic dynamics can reasonably be assumed to be overwhelmed by the Lévy statistics of the autonomic input and therefore ignored in the model. The deterministic force is therefore modeled by the cooperative behavior of the pacemaker cells.

We present a detailed model of the cooperative behavior of the sinoatrial cells, based on the micro-dynamics of a complex network elsewhere, but here we restrict our analysis to a hypothetical control model that is consistent with such networking ideas.

### 3.1. Linear Control

Linear dissipation has a long pedigree in the physical sciences, including the fluctuation-dissipation relation obtained by Einstein in his 1905 paper on classical diffusion. He was able to relate the temperature of the ambient fluid to the ratio of the strength of the random force, buffeting the diffusing particle, and the linear dissipation parameter. This ratio constitutes the first fluctuation-dissipation theorem and it was over a century before this physical theorem was systematically interpreted in terms of the control theory concept (Sandberg et al., 2011) of negative feedback.

For the inter-beat interval dynamics the simplest non-trivial deterministic driver is linear and does represents a negative feedback process. In this case Equation (4) reduces to a linear stochastic differential equation, which in the physics literature goes by the name of the linear Langevin equation:

(5) $$\begin{array}{r} dI\left(t\right)=-\lambda_{0}I\left(t\right)dt+d\xi \;(t). \end{array}$$

For a diffusing particle the fluctuations inject energy from the ambient fluid into the particle motion and the linear dissipation parameter quantifies the rate of absorption of energy back into the ambient fluid, thereby maintaining an energy balance, on average. The formal solution to Equation (5) is given by

(6) $$\begin{array}{r} I\left(t\right)=e^{-\lambda_{0}t}I\left(0\right)+e^{-\lambda_{0}t}\overset{t}{\underset{0}{\int }}d\xi \;(t^{\prime })e^{\lambda_{0}t^{\prime }}. \end{array}$$

The statistical properties of the solution to the dynamic equation are straight forward to determine, when the random driver is a Wiener process; noise that is delta correlated in time, with Gaussian statistics. These are the statistical assumptions typically made in physical systems.

However, the historical statistical assumptions are not appropriate for modeling HRV, with the stochastic driver being the result of competing neuroautonomic inputs. Based on the earlier discussion, we assume *dξ*(*t*) to be a delta correlated Lévy stable and not a Wiener process. The analysis relating the solution to the dynamic equation and the corresponding PDF was carried out (West and Seshadri, 1982) for *I*(0) ≠ 0, but we do not need that level of generality here and so we simplify things and set *I*(0) = 0 and relegate the details of the supporting analysis to Appendix 5.2.

In Appendix 5.2 the dynamic variable *I*(*t*) is shown to have a probability P(I,t)dI of being in the interval (I,I+dI) at the time *t*. The kinetic equation for the evolution of the PDF corresponding to the linear Langevin equation is shown to be given by the fractional Fokker-Planck equation (FFPE):

(7) $$\begin{array}{r} \frac{\partial P\left(I,t\right)}{\partial t}=\frac{\partial }{\partial I}\left[\lambda_{0}IP\left(I,t\right)\right]+\sigma^{2}\partial_{\left|I\right|}^{\alpha }\left[P\left(I,t\right)\right], \end{array}$$

which differs from Equation (2) in the Introduction, through the inclusion of the linear feedback term. The solution to the FFPE is the Lévy PDF (West and Seshadri, 1982):

(8) $$\begin{array}{r} P\left(I,t\right)=\frac{1}{\pi }\underset{0}{\overset{\infty }{\int }}d\omega \cos\left(I\omega \right)\exp\left[-\sigma_{\alpha }^{2}\left(t\right)\omega^{\alpha }\right], \end{array}$$

with a time-dependent “width”:

$$\begin{array}{l} \sigma_{\alpha }^{2}\left(t\right)=\frac{\sigma^{2}}{\lambda_{0}\alpha }\left(1-e^{-\alpha \lambda_{0}t}\right). \end{array}$$

This PDF becomes equivalent to the translationally invariant Lévy distribution discussed in the Introduction when λ_0_ = 0, in which case

(9) $$\begin{array}{r} \underset{\lambda_{0}\to 0}{\lim}\sigma_{\alpha }^{2}\left(t\right)=\sigma^{2}t, \end{array}$$

and the constant coefficient is identified with the diffusion coefficient *K*_α_.

**Figure 2:**
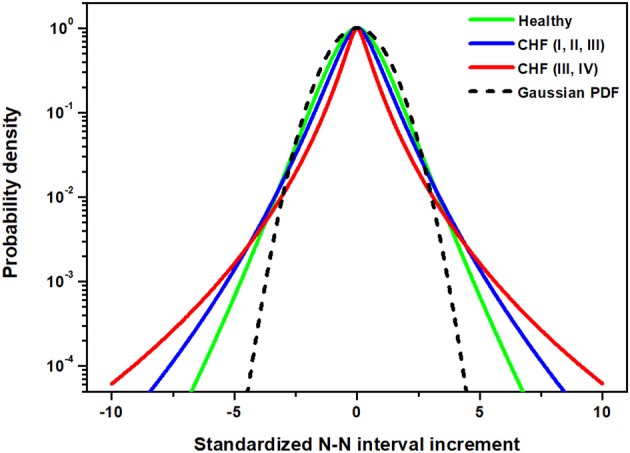
The PDF approximated by the non-Gaussian model using Equation (22) in Appendix with parameter λ being the group average listed in Appendix 5.1 for three studied groups: healthy individuals, CHF patients of class I, II, III and CHF patients of class III, IV.

The above argument was used (West and Deering, 1994) to explain the earlier results (Peng et al., 1993), in the asymptotic case

(10) $$\begin{array}{r} \underset{t\to \infty }{\lim}\sigma_{\alpha }^{2}\left(t\right)=\frac{\sigma^{2}}{\lambda_{0}\alpha }, \end{array}$$

where the Lévy PDF becomes time independent, but with a strength parameter different from that characterizing the stochastic driver. However, this argument alone cannot explain the data depicted in Figure 2. The analysis must be generalized to include the natural physiologic control necessary to avoid unphysiologically large fluctuations in HRV, which are a hallmark of pathological condition.The expectation here is that an additional dissipative tempering of the Lévy fluctuations attenuates the extremes observed in the HRV PDF of severe CHF patients depicted in Figure 2. Let us examine this last conjecture more closely.

### 3.2. Nonlinear Control

There is no á *priori* reason to restrict the analysis of HRV dynamics to linear negative feedback. In physical systems the fluctuations and linear dissipation have a common origin and consequently are tied together by a fluctuation-dissipation relation. In biological systems, however, the dissipation and the random force often have different sources and the feedback must adapt to regulate the properties of the dynamic response to the random force. In the case of HRV the deterministic force is here assumed to be given by the collective behavior of the sinoatrial pacemaker cells. Such cooperative behavior has been shown to be generic for large classes of complex networks including members of the Ising universality class (West, 2014a). One such model near criticality is adopted below to describe HRV dynamics.

A generic version of Equation (4) was analyzed by Chechkin et al. (2005) in which the deterministic term was gvien by the nonlinear force −λ(*I*)*I* :

(11) $$\begin{array}{r} dI\left(t\right)=-\lambda \left(I\right)\;I\;\left(t\right)+d\xi \;(t). \end{array}$$

Equation (11) reduces to the linear Langevin equation of the last section with λ_0_ ≡ λ(0) and *dξ* (*t*) is again assumed to be a stable Lévy process. In general the feedback term is a polynomial, but we restrict the analysis to the largest-order term, since this is the term that dominates the control process and write

(12) $$\begin{array}{r} \lambda \left(I\right)\approx \lambda_{2n}I^{2n}, \end{array}$$

where λ_2*n*_ > 0 and *n* is an integer. Note the index is restricted to even order to ensure that higher-order terms to not change the symmetry of the control process from that of the linear feedback term.

Note further that the sinoatrial node is comprised of a complex network of interacting pacemaker cells, each with its own internal clock, which acts within the collective to coordinate the heart beat rhythm. A number of mathematical models have been devised that manifest such collective behavior, providing insight into critical phase transitions in non-physical networks. One such model is the Decision Making Model (DMM) (West et al., 2014), which near criticality reduces to the cubic Langevin equation:

(13) $$\begin{array}{r} dI\left(t\right)=-\lambda_{0}I\left(t\right)-\lambda_{2}I^{3}\left(t\right)+d\xi \;(t). \end{array}$$

In the DMM the linear term has the generic property of going to zero, λ_0_ → 0, as the critical point is approached by adjusting a control parameter to its critical value. In the DMM *n* = 1 and despite the input driver being a Lévy process, the inclusion of a nonlinear term of order *n* = 1 quenches the divergence of 〈*I*(*t*)^2^〉 (West and Seshadri, 1982; Chechkin et al., 2005) and the brackets denote an average over an ensemble of realizations of the random driver.

Of course this investigation would be of little interest if it depended on the details of the interaction within the network. This is not the case with critical phenomena. Networks that undergo phase transitions manifest scaling, which results from the universality of the dynamics, and this is manifest in the fractal nature of HRV. Consequently the approximate form of the dynamic equations in the vicinity of the critical point is also universal, see Landau's discussion of critical slowing down when a fluid transitions from laminar to turbulent flow (Landau and Lifshitz, 1987). This is the kind of control processes of interest to us for the HRV data. Note, however, that higher-order diverging moments would require equally higher-order feedback terms to control the process.

It is possible to use the technique presented in Appendix 5.2 to replace the Langevin equation containing the highest-order nonlinear term in the dissipation, with a corresponding FFPE. We present the details of the analysis in Appendix 5.3 to obtain

(14) $$\begin{array}{r} \frac{\partial P\left(I,t\right)}{\partial t}=\frac{\partial }{\partial I}\left[\lambda_{2n}I^{2n+1}P\left(I,t\right)\right]+\sigma^{2}\partial_{\left|I\right|}^{\alpha }\left[P\left(I,t\right)\right], \end{array}$$

the same FFPE studied by Checkin et al., where, as they point out, the nonlinear coefficient has the role of a confining potential. In a similar way, West et al. (2014) make the association of the cubic term in the Langevin equation (*n* = 1) with a bimodal potential, resulting from the collective behavior of the DMM network at criticality. In the latter case *I*(*t*) would correspond to the global variable, that being the average over all the elements of the network.

Of course, we cannot solve Equation (14) in general, but it is not necessary to have the exact solution in hand to obtain the information we need to test our control hypothesis. The asymptotic behavior of the steady-state solution is sufficient. Consider the equation for the steady-state PDF

(15) $$\begin{array}{r} P_{ss}\left(I\right)=\underset{t\to \infty }{\lim}P\left(I,t\right) \end{array}$$

given by

(16) $$\begin{array}{r} \frac{\partial }{\partial I}\left[\lambda_{2n}I^{2n+1}P_{ss}\left(I\right)\right]+\sigma^{2}\partial_{\left|I\right|}^{\alpha }\left[P_{ss}\left(I\right)\right]=0. \end{array}$$

We put the Reisz-Feller fractional derivative in the convenient form

(17) $$\begin{array}{r} \partial_{\left|I\right|}^{\alpha }\left[P_{ss}\left(I\right)\right]=\frac{1}{\pi }\text{Γ}\left(\alpha +1\right)\sin\left[\pi \alpha /2\right]\underset{-\infty }{\overset{\infty }{\int }}\frac{P_{ss}\left(I^{\prime }\right)dI^{\prime }}{|I-I^{\prime }{|}^{1+\alpha }}, \end{array}$$

which allows us to analyze the solution to Equation (16) from the equivalent form of the Reisz-Feller fractional derivative:

(18) $$\begin{array}{r} \partial_{\left|I\right|}^{\alpha }\left[P_{ss}\left(I\right)\right]=-{\text{C}}_{\alpha }\frac{d^{2}}{dI^{2}}\underset{-\infty }{\overset{\infty }{\int }}\frac{P_{ss}\left(I^{\prime }\right)dI^{\prime }}{|I-I^{\prime }{|}^{\alpha -1}}, \end{array}$$

with the coefficient

$$\begin{array}{l} C_{\alpha }=\frac{\sigma^{2}}{2\text{Γ}\left(2-\alpha \right)\cos\left(\alpha \pi /2\right)}. \end{array}$$

Inserting Equations (18) into (16) and assuming zero flux boundary conditions yields the integral equation for the steady-state PDF (Chechkin et al., 2005):

(19) $$\begin{array}{r} \lambda_{2n}I^{2n+1}P_{ss}\left(I\right)-C_{\alpha }\frac{d}{dI}\underset{-\infty }{\overset{I}{\int }}\frac{P_{ss}\left(I^{\prime }\right)dI^{\prime }}{|I-I^{\prime }{|}^{\alpha -1}}=0. \end{array}$$

Here again we cannot obtain an exact result, so we estimate the value of the integral asymptotically in Appendix 5.3.

**Figure 3:**
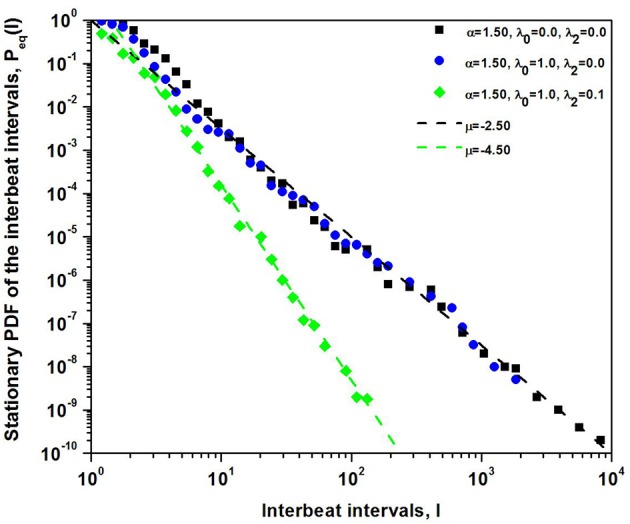
The numerical integration of the nonlinear Langevin equation is used to give the histograms for the PDF with the Lévy index α = 1.5. The computational results are fit extremely well by the steady-state IPL PDF with IPL index μ = α + 2*n* + 1 for *n* = 0 and *n* = 1.

The approximate asymptotic steady-state solution to Equation (14) is given by the solution to Equation (19) to be the IPL PDF (Chechkin et al., 2005):

(20) $$\begin{array}{r} P_{ss}\left(I\right)=\frac{N_{\mu }}{|I{|}^{\mu }}\;\text{;}\;\mu =\alpha +2n+1, \end{array}$$

where *N*_μ_ is the normalization constant. In Figure 3 the steady-state PDF given by the solution to the FFPE is the IPL (Equation 20). The steady-state PDF is fit to the numerical integration of the nonlinear Langevin equation. In the calculation a Lévy process with α = 1.5 is used to drive the dynamics of the sinoatrial node modeled as a DMM network near the tipping point. The deterministic force is selected to have either *n* = 0 or *n* = 1, or both. It is clear from the figure that the formal solution captures the IPL form predicted by the steady-state solution to the FFPE with either μ = 2.5 or μ = 4.5.

It is well known that for an IPL index μ ≥ 3 the second moment $\langle I^{2}\rangle$ is finite, implying that the central limit theorem holds asymptotically. Consequently, for *n* = 1 the statistics of *I*(*t*) have a finite second moment asymptotically and the steady-state PDF asymptotically transitions from Lévy stable to Gaussian. This transition explains the results obtained by Kiyono et al. (2004, 2006), without the need to exponentially temper the Lévy driver, relying solely on the sinoatrial dynamics to temper the extreme fluctuations resulting from the competition between the two branches of the involuntary nervous system.

## 4. Discussion and Conclusions

A quarter century ago we (Goldberger et al., 1990) hypothesized that disease is associated with the loss of complexity, which has been subsequently tested using HRV data. One example, in support of this hypothesis, is a study of combat casualties involving 70 acutely injured adults, in a U.S. Army Combat Support Hospital (CSH) located at Ibn Sina Hospital, Baghdad, Iraq, during the recent conflict. This study determined that the complexity of HRV dynamics over a range of time scales was lower in high-risk than in low-risk combat casualties (Cancio et al., 2013), using multi-scale entropy (MSE) as a measure of complexity. It had been determined earlier (Costa et al., 2008) that the scaling of the data established that the MSE measure of complexity was higher for healthy subjects than for those with congestive heart failure or with atrial fibrillation. The greater complexity for healthy individuals only became evident beyond a certain level of coarse graining of HRV data.

Struzik et al. (2008) emphasize an interpretation in which healthy heart rate represents the upper bound on HRV, and reduced variability of heart rate fluctuations is of clinical risk. They call into question the complexity hypothesis and its clinical interpretation. Similar observations have been made in an extensive study of Stein et al. (2005). In particular, they find that there is an increase in fluctuations, which they interpret as an increase in complexity, of heart rate in chronic-heart failure patients, specifically those at risk of death, just as those observed in Figure 2. Thus, it would appear from the figure that complexity has increased in those patients that are the more severely diseased in contradiction to the complexity hypothesis. This interpretation adopts the perspective that complexity is proportional to variability and therefore is greatest in physiological processes with diverging central moments. A more thoughtful analysis of complexity reveals something different.

The analysis of HRV data by Peng et al. (1993) indicated that the statistics of the changes in heart beat intervals are Lévy stable. The Lévy statistics have the undesirable property that the second moment of heart beat time intervals diverge and consequently implies that the hear beat of a healthy person would be asymptotically unstable. This instability was shown to be quenched by a number of investigators (Kiyono et al., 2004; Hayano et al., 2011), through the introduction of a tempered Lévy driver.

The data shown in Figure 2 suggest the use of exponentially tempered Lévy statistics to explain the difference between the healthy individuals and CHF patients. The tempering control mechanism would suppress the largest excursions in time, which persist in the severe CHF group. The pathophysiology of the HRV PDFs being Lévy stable would then be the result of the suppression of a physiological control mechanism as suggested in an earlier publication (West, 2014b). However, the form of this earlier control mechanism would need to operate at the level of the involuntary nervous system.

The scaling of the statistics for the central or Lévy part of the tempered PDF is the same for both the healthy and CHF patients in Figure 2. However, there is the additional scaling of the HRV in the tempered Lévy PDF produced by the criticality of the healthy sinoatrial node dynamics. Criticality, as manifest in the cubic term in the Langevin equation, reduces the span of the fluctuations from those that persist in severe CHF condition to those present in more manageable CHF cases. This second scaling, which is produced by the proposed control mechanism, certainly adds to the overall complexity of the process, by introducing a second scaling mechanism. It is the loss of this control that lifts the restriction on the span of fluctuations in HRV time series, resulting in cardiac pathologies. Consequently, those that suffer from a serious cardiological condition have greater heart rate variability, but their HRV is certainly less complex than those whose HRV variability is restricted by the nonlinear control process. Thus, interpreting complexity to be proportional to the second moment (variability) in the HRV context is too simplistic.

Complementary observations can be made through the analysis of the HRV with the help of detrended fluctuation analysis. The work of Ivanov et al. (1999) and Amaral et al. (2001) have demonstrated reduced multifractality associated with impaired parasympathetic control and pathological condition of CHF, respectively. This corresponds to a reduction of long-range correlations present in the HRV of healthy individuals, an outcome related to a loss of dynamical properties of the cardiovascular system. However we should point out that despite semantic similarities, the scaling exponent of DFA quantifies temporal properties of HRV thought to be statistical fractal time series. Contrarily, the control mechanism introduced herein is presented in terms of a fractional Fokker-Planck equation (Equation 14), which leads to investigations of HRV through its PDF distribution. The methods are thus complementary, but not directly related, as DFA characterizes temporal correlations present in random walk-like process, while HRV control framework focuses on the steady-state distribution of such random walk time series. Additionally we need to point out that the DFA is a method of data processing of individual time series that assumes the central moments of the time series are sufficient to characterize the process. The fractional Fokker-Planck equation (FFDE) makes no such assumption. It is a theoretical model that describes the evolution of the probability density function for the physiologic process and its solution captures the full dynamics of an ensemble of time series. When the DFA scaling parameter is equal to that obtained from the second moment calculated using the solution to the FFPE, the theoretical model satisfies the assumptions necessary for the DFA. When the two do not agree it suggests that the dynamics are determined by more than central moment properties.

The adaptive feedback control process produced by the collective behavior of the pacemaker cells, postulated herein, is physiologically simpler than the exponential tempering suggested earlier (West, 2014b) and applying Occam's razor, is the more likely explanation. The nonlinear potential is a collective effect within the sinoatrial node dynamics and produces a tempered response to the stochastic excitations. This tempering of the extreme HRV excursions is pathologically suppressed within the severe CHF group. The pathophysiology of the HRV PDF being Lévy stable is then the result of a suppressed control process, which can no longer determine the maximum size of the inter-beat intervals, resulting in loss of control and disease.

An explanation of the source of the nonlinear control mechanism based on the sinoatrial node dynamics being poised at criticality is presently being developed. This was introduced in the text as a conjecture and will be discussed in detail in a future publication.

## Data Availability

Publicly available datasets were analyzed in this study. This data can be found here: https://physionet.org.

## Author Contributions

BW developed the theoretical formalism. MT performed numerical simulations. Both authors discussed the results and contributed to the final manuscript.

### Conflict of Interest Statement

The authors declare that the research was conducted in the absence of any commercial or financial relationships that could be construed as a potential conflict of interest.
