# Supplementary material for: Hypothetical Control of Heart Rate Variability
Source: Front Physiol. 2019 Aug 23;10:1078. doi: 10.3389/fphys.2019.01078 (PMC6716055; doi:10.3389/fphys.2019.01078)
Supplement: Supplementary file 1 [file Presentation_1.pdf]

5 APPENDIX

5.1 Materials and methods

5.1.1 Data

All data used were from the MIT-BIH RR Interval Database (Goldberger et al., 2000).The Normal Sinus Rhythm RR Interval Database was used as the healthy control group. This database included 54 long-term RR interval recordings of subjects in normal sinus rhythm. The Congestive Heart Failure RR Interval Database (NYHA class I, II and III) and the BIDMC Congestive Heart Failure Database (NYHA class III and IV) were used as the pathological groups. Those databases included 29 and 15 long-term RR interval recordings, respectively. The original ECG signals in all cases were digitized at 128 Hz and the beat annotations were obtained by automated analysis with manual review and correction. Inclusion of NYHA patients of class III in both CHF groups is a consequence of analyzing publicly available datasets and not an intentional choice. Available RR time series are anonymized, the information about the NYHA class of individual patients is not available and more precise separation is not possible. As a result both CHF groups include patients of NYHA class III.

5.1.2 Linear analysis

The mean heart rate, standard deviation of N-N intervals (SDNN) and root mean square of successive differences of N-N intervals (rMSSD) were used as time-domain measures of HRV (Shaffer and Ginsberg, 2017). The power spectrum densities were estimated by Welch's averaged periodogram method applied to R-R time series resampled at 2 Hz with the use of a cubic spine interpolation. Very low frequency power (VLF, 0.0033 to 0.04 Hz), low frequency (LF, 0.05-0.15 Hz) and high frequency power (HF, 0.15-0.40 Hz) were calculated from the entire 24-hour segments.

5.1.3 Nonlinear analysis

Detrended Fluctuation Analysis (DFA) quantifies fractal-like correlations in time series (Peng et al., 1995b). The scaling exponent DFA α indicates the scaling of root-mean-square fluctuations of the integrated and detrended HRV data as a function of observation window of increasing size (Peng et al., 1995a). Parameter α_1 captures brief fluctuations (4-11 beats), while value of α_2 describes long-term scaling (>11 beats).

5.1.4 Non-Gaussianity index

The analysis of non-Gaussianity of HRV starts from integrating and detrending (third order polynomial for the trend was used herein) R-R interval time series (Kiyono et al., 2004, 2006). After the detrending procedure heart rate increments at scale s are defined as $\Delta_s B(t) = B^*(t+s) - B^*(t)$, where $B^*(t)$ is a deviation of the HRV from the polynomial trend. Then the PDF of differences $\Delta_s B(t)$ normalized by the SD is evaluated. Finally, the non-Gaussianity index λ_s is estimated as

$$\lambda_s = \sqrt{\frac{2}{q(q-2)}\left[\ln(\frac{\sqrt{\pi}\left\langle|\Delta_s B|^q\right\rangle}{2^{q/2}}\right) - \ln\Gamma\left(\frac{q+1}{2}\right)\Bigg]}, \tag{21}$$

where $\left\langle|\Delta_s B|^q\right\rangle$ denotes an estimated value of the q-th order absolute moment of $[\Delta_s B]$. Following (Hayano et al., 2011) we calculate λ_s based on the $1/4$th order moment $(q = 0.25)$ and we set the scale s at 25 s. The parameter λ_s was originally introduced as a parameter of log-normal cascade model describing

non-Gaussian distributions in the study of intermittency of hydrodynamic turbulence (Castaing et al., 1990):

$$P_{\lambda,\sigma_0}(x) = \int_0^{\infty} \frac{1}{\sqrt{2\pi}\lambda} \exp\left(-\frac{\ln^2(\sigma/\sigma_0)}{2\lambda^2}\right) \times \frac{1}{\sqrt{2\pi}\sigma} \exp\left(-\frac{x^2}{2\sigma^2}\right) d(\ln\sigma). \tag{22}$$

The value of λ_s close to zero corresponds to a PDF close to a Gaussian distribution, while a larger value of λ_s means that the observed PDF has fatter tails and a sharper peak in comparison with the Gaussian distribution.

5.1.5 Results

The values (mean ± SD) of listed HRV indices for the group of healthy subjects and two groups of patients with CHF are presented in Table 5.1. All indices overlap with values reported in literature for comparable datasets (Shaffer and Ginsberg, 2017; Beckers et al., 2006; Hayano et al., 2011). In line with previous studies (Hayano et al., 2011), congestive heart failure leads a decrease in values of numerous HRV indices. We observe that the power of the HRV in LF and HF frequency bands, LF/HF ratio or scaling coefficients estimated through DFA are affected by the pathological condition. This corresponds to a change in sympatho-vagal balance, where the CHF leads to a dominance of parasympathetic branch of the ANS. Reduced DFA scaling coefficients reflect diminished correlations present in HRV time series, reflecting reduced complexity of cardiovascular control mechanisms.

5.2 FFPE

The conditional PDF $P(\mathcal{I},t) \equiv P(\mathcal{I},t\,|\mathcal{I}_0, t=0)$ is the average of the phase space density function $\rho(\mathcal{I},t)$ over an ensemble of realizations of $\xi(t)$:

$$P(\mathcal{I},t) \equiv \langle\rho(\mathcal{I},t)\rangle_{\xi}\,. \tag{23}$$

The phase space density function is defined as

$$\rho(\mathcal{I},t) = \delta(\mathcal{I} - I(t))\,, \tag{24}$$

where $I(t)$ is the solution to the Langevin equation for a particular realization of $d\xi(t)$, in this case given by Eq.(6). The formal expression for the PDF is then given by

$$P(\mathcal{I},t) \equiv \langle\delta(\mathcal{I} - I(t))\rangle_{\xi}\,. \tag{25}$$

The evolution of phase space density function is determined by the Liouville equation, or continuity equation

$$\frac{\partial\rho(\mathcal{I},t)}{\partial t} + \frac{\partial}{\partial\mathcal{I}}\left[\dot{I}(t)\rho(\mathcal{I},\iota)\right] = 0. \tag{26}$$

The function $\dot{I}(t)$ in the continuity equation is defined by differentiating the solution Eq.(6) with respect to time:

$$\dot{I}(t) = -\lambda_0 I(t) + e^{-\lambda_0 t}\frac{d}{dt}\int_0^t d\xi\left(t'\right)e^{\lambda_0 t'}, \tag{27}$$

where for notational ease we introduce the function

$$f(t) \equiv e^{-\lambda_0 t} \frac{d}{dt} \int_0^t d\xi \left(t'\right) e^{\lambda_0 t'} \tag{28}$$

to replace the integral term. The Fourier transform of $\rho\left(\iota, t\right)$ is

$$\phi\left(\omega, t\right) = \int_0^\infty \cos\left(i\omega\right) \rho\left(i, t\right) di \tag{29}$$

so that the continuity equation can be written as

$$\frac{\partial \phi\left(\omega, t\right)}{\partial t} + \lambda_0 \omega \frac{\partial \phi\left(\omega, t\right)}{\partial \omega} = i\omega f(t) \phi\left(\omega, t\right). \tag{30}$$

To solve Eq.(30) we introduce the deterministic operator

$$\mathcal{L}_0 \equiv -\lambda_0 \omega \frac{\partial}{\partial \omega}, \tag{31}$$

the stochastic operator

$$\mathcal{L}_1\left(t\right) \equiv i\omega f(t), \tag{32}$$

and rewrite Eq.(30) as

$$\frac{\partial \phi\left(\omega, t\right)}{\partial t} = \left[\mathcal{L}_0 + \mathcal{L}_1\left(t\right)\right] \phi\left(\omega, t\right). \tag{33}$$

The solution to this equation is given by

$$\phi\left(\omega, t\right) = e^{\mathcal{L}_0 t} \exp\left[j\omega \int_0^t d\xi \left(t'\right) e^{\lambda_0 t'} \right] \;\; ; \;\; j = \sqrt{-1} \tag{34}$$

and the mathematical details are given by West and Seshadri (West and Seshadri, 1982).

The function $\phi\left(\omega, t\right)$ can be averaged over an ensemble of realizations of $d\xi\left(t\right)$ to yield the characteristic function $\widehat{P}\left(\omega, t\right)$:

$$\widehat{P}\left(\omega, t\right) = \left\langle \phi\left(\omega, t\right) \right\rangle_\xi = e^{\mathcal{L}_0 t} \left\langle \exp\left[j\omega \int_0^t d\xi \left(t'\right) e^{\lambda_0 t'} \right] \right\rangle_\xi, \tag{35}$$

which is the Fourier transform of the PDF. For an arbitrary analytic function $g(\tau)$, Doob (Doob, 1942) showed that for a differential Lévy process $dB(\tau)$ of strength σ^2, with an index α and which is delta correlated in time:

$$\left\langle \exp\left(\int_0^t jg(\tau) dB(\tau) \right) \right\rangle_B = \exp\left(-\sigma^2 \int_0^t \left| g(\tau) \right|^\alpha d\tau \right). \tag{36}$$

Consequently, if we assume the stochastic driver $d\xi(t)$ is such a Lévy process, after further analysis (West and Seshadri, 1982), the equation of motion for the characteristic function is determined to be

$$\frac{\partial \widehat{P}(\omega,t)}{\partial t} + \lambda_0 \omega \frac{\partial \widehat{P}(\omega,t)}{\partial \omega} = -\sigma^2 \left|\omega\right|^{\alpha} \widehat{P}(\omega,t), \tag{37}$$

which is solved (West and Seshadri, 1982) to obtain

$$\widehat{P}(\omega,t) = \exp\left[-\sigma_{\alpha}^{2}(t)\left|\omega\right|^{\alpha}\right], \tag{38}$$

where the time-dependent 'width' of the distribution is

$$\sigma_{\alpha}^{2}(t) = \frac{\sigma^2}{\lambda_0 \alpha}\left(1 - e^{-\alpha\lambda_0 t}\right). \tag{39}$$

The PDF generated by the linear Langevin equation is therefore

$$P(\mathcal{I},t) = \frac{1}{\pi}\int_{0}^{\infty} d\omega \cos(\mathcal{I}\omega) \exp\left[-\sigma_{\alpha}^{2}(t)\,\omega^{\alpha}\right], \tag{40}$$

a symmetric Lévy PDF.

5.3 Steady-state solution

Chechkin *et al.* (Chechkin et al., 2005) argue that the behavior of the steady-state PDF can be determined from an analysis of the contributions to the integral in Eq.(19) in the vicinity of the pole. In particular, the dominant contribution to the integral arises from the region to the left of the pole $\mathcal{I} = \mathcal{I}'$ and consequently the operator may be approximated by

$$\lambda_{2n}\mathcal{I}^{2n+1}P_{ss}(\mathcal{I}) - C_{\alpha}\frac{d}{d\mathcal{I}}\int_{-\infty}^{\mathcal{I}} \frac{P_{ss}(\mathcal{I}')d\mathcal{I}'}{\left|\mathcal{I}-\mathcal{I}'\right|^{\alpha-1}} = 0. \tag{41}$$

The integral term may be expressed to the lowest-order approximation

$$\int_{-\infty}^{\mathcal{I}} \frac{P_{ss}(\mathcal{I}')d\mathcal{I}'}{\left|\mathcal{I}-\mathcal{I}'\right|^{\alpha-1}} = \mathcal{I}^{1-\alpha}\int_{-\infty}^{\mathcal{I}} \frac{P_{ss}(\mathcal{I}')d\mathcal{I}'}{\left|1-\frac{\mathcal{I}'}{\mathcal{I}}\right|^{\alpha-1}}$$

so that expanding the numerator and noting

$$\int_{-\infty}^{\mathcal{I}} P_{ss}(\mathcal{I}')d\mathcal{I}' \approx 1$$

yields to the lowest-order approximation

$$\int_{-\infty}^{\mathcal{I}} \frac{P_{ss}(\mathcal{I}')d\mathcal{I}'}{|\mathcal{I}-\mathcal{I}'|^{\alpha-1}} \backsim \mathcal{I}^{1-\alpha}.$$

Inserting this expression for the integral into Eq.(41) and taking the derivative yields, after some algebra, the asymptotic symmetric steady-state PDF:

$$P_{ss}\left(\mathcal{I}\right) = \frac{N_{\mu}}{|\mathcal{I}|^{\mu}} \;\; ; \; \mu = 2n + \alpha + 1, \tag{42}$$

with the normalization constant N_{μ}.
